# Right-Sided Fourth Branchial Cleft Cyst in a Neonate: A Case Report and Literature Review

**DOI:** 10.7759/cureus.71624

**Published:** 2024-10-16

**Authors:** Osama Qumsieh, Maram Heeh, Anas Abukhalaf, Kenana Altel, Fatima Amer, Sajeda Nawajaa

**Affiliations:** 1 Pediatric Surgery, Palestine Polytechnic University, Hebron, PSE; 2 Pediatric Surgery, Al-Ahli Hospital, Hebron, PSE; 3 Pediatrics, Al-Ahli Hospital, Hebron, PSE; 4 General Surgery, Al-Ahli Hospital, Hebron, PSE; 5 Pediatric Surgery, Hebron University, Hebron, PSE

**Keywords:** anomalies, compression, fourth branchial cleft cysts, mediastinum, neonate, rare, surgical excision

## Abstract

Anomalies of the fourth branchial cleft are exceedingly uncommon, presenting with a diverse array of clinical manifestations. The majority of branchial cleft anomalies, approximately 95%, are of the second type, with a mere 2% attributed to the fourth type. The latter is notably more prevalent on the left side, with reports indicating an 85% incidence. Herein, we report the case of a neonate presenting with a right-sided fourth branchial cleft cyst, which manifested at birth and subsequently underwent progressive enlargement, resulting in airway and esophageal compromise. The management strategy involved surgical excision, and the definitive diagnosis was established through histopathological examination, underscoring the rarity and diagnostic challenge of this form of branchial cyst.

## Introduction

Branchial cleft anomalies, thyroglossal duct cysts, and vascular malformations constitute the prevalent congenital masses of the neck with branchial cleft cysts (BCCs), accounting for approximately 30% of cases and typically manifesting during childhood or early adulthood [1]. The embryonic branchial apparatus, which includes five pairs of arches with a cartilaginous core (mesoderm), is separated externally by four paired branchial clefts (ectoderm) and internally by four paired pharyngeal pouches (endoderm), eventually developing into the principal structures of the head and neck [2]. The regression of the clefts is complete by the seventh week of gestation [3]. Consequently, the improper obliteration of these clefts results in the formation of cysts, sinus tracts, fistulas, or cartilaginous remnants within the neck region [4]. These anomalies are typically lined with either respiratory or squamous epithelium [5] and may exhibit an autosomal dominant pattern of inheritance [4]. Among branchial cleft anomalies, second BCCs are the most prevalent, constituting approximately 95% of cases, followed by first BCCs (5-25%) [3,6] and third BCCs (2-8%) [3]. First BCCs are characterized by swelling in the submandibular region, posterior or anterior to the earlobe, whereas second, third, and fourth BCCs are located in the supraclavicular area. The presence of a fistula or sinus may reveal cutaneous openings at the junction of the lower and middle thirds of the anterior sternocleidomastoid muscle [7]. Fourth BCCs, representing the rarest type (2%), have a less well-defined course due to their infrequency [8]. These cysts are situated anterior to the aortic arch on either side, within the mediastinum in the tracheoesophageal groove [9]. The tract encircles either the subclavian artery or the aortic arch, depending on the side, and ascends to a loop over the hypoglossal nerve, terminating at the apex of the pyriform sinus. Such anomalies can manifest either in isolation or as components of broader developmental syndromes [10,11]. Understanding fourth BCCs is significant and crucial for several reasons. Clinically, these cysts are rare and can be challenging to diagnose due to their atypical presentation and deep location in the neck. Early and accurate identification is crucial to prevent complications such as infection, abscess formation, or difficulty breathing. Additionally, misdiagnosis can lead to inappropriate treatment. Knowledge of these cysts also aids in understanding developmental anomalies of the branchial apparatus, which can be associated with other congenital syndromes, helping to inform both surgical planning and long-term management. In this report, we present a 27-day-old female neonate who exhibited intermittent grunting, frothy oral secretions, and cyanosis during feeding from the age of six days. Additionally, she had an enlarging right-sided neck swelling since birth, leading to the diagnosis of a right fourth BCC, which was successfully treated with surgical intervention. This case study aims to shed light on this uncommon type of BCC.

## Case presentation

A 27-day-old female neonate, delivered at term (GA: 40+3) through normal delivery, presented with intermittent grunting and frothy oral secretions starting at six days of age. Additional symptoms included cyanosis, respiratory distress, feeding difficulties, and a progressively enlarging right neck swelling noted since birth, which was concerning to the parents.

On physical examination, a large, hard, non-tender mass approximately 3×3 cm in size was observed in the right carotid triangle area. The mass was negative for transillumination and did not exhibit skin discoloration, visible veins, or discharge. The neonate displayed dysmorphic features, including micrognathia, a low anterior hairline, a hairy forehead, long thick eyelashes, synophrys, arched eyebrows, a long smooth philtrum, a thin upper lip, and low-set posteriorly rotated ears. She also exhibited growth retardation, with growth parameters recorded as follows: weight: 2.6 kg, head circumference: 32 cm, and height: 49 cm.

**Figure 1:**
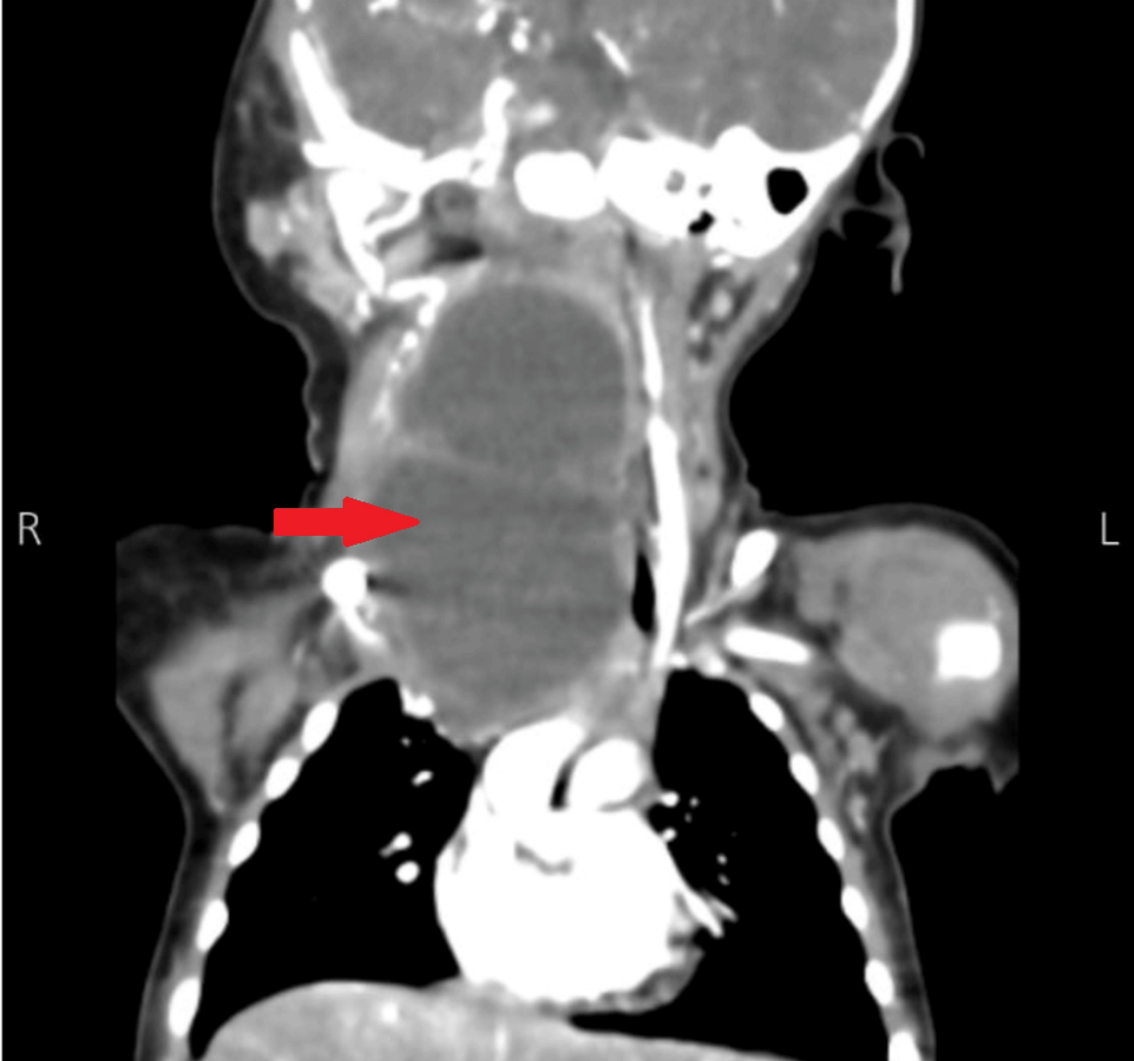
The coronal section of the contrast-enhanced CT scan shows 3.5×3.1×5.6 cm of cervical mass extending to the mediastinum, shifting cervical structures, and causing a mass effect (red arrow). CT: computed tomography

**Figure 2:**
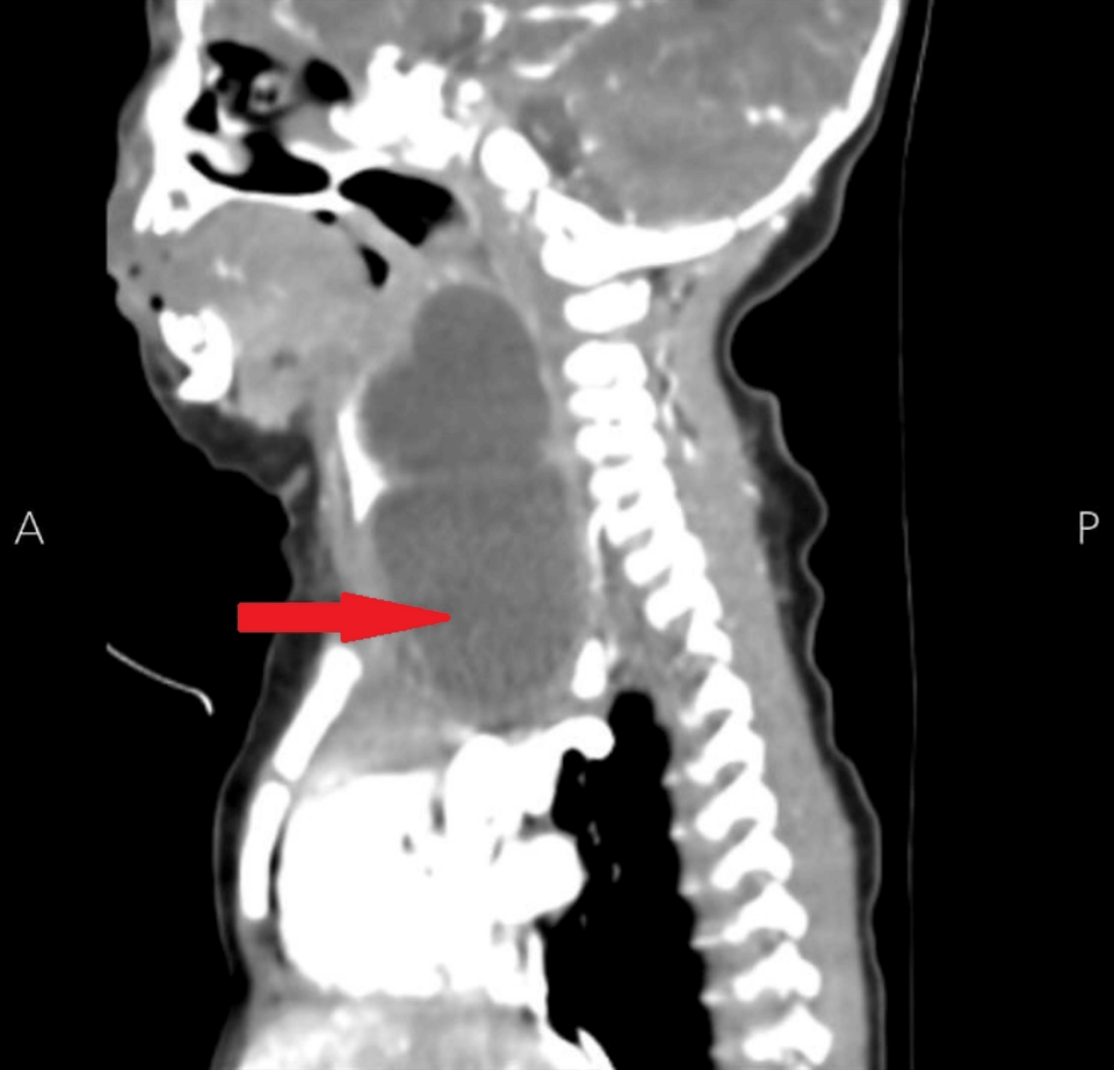
The sagittal section of the contrast-enhanced CT scan shows 3.5×3.1×5.6 cm of cervical mass extending to the mediastinum, shifting cervical structures, and causing a mass effect (red arrow). CT: computed tomography

Ultrasound imaging of the right neck swelling revealed a right upper mediastinal mass with cystic and solid components measuring approximately 8 cm, extending to the right side of the neck with thick septation and intracystic fluid. Computed tomography (CT) scans confirmed the presence of a right cervical cystic lesion measuring 3.5×3.1×5.6 cm, extending into the mediastinum (Figure 1 and Figure 2).

**Figure 3:**
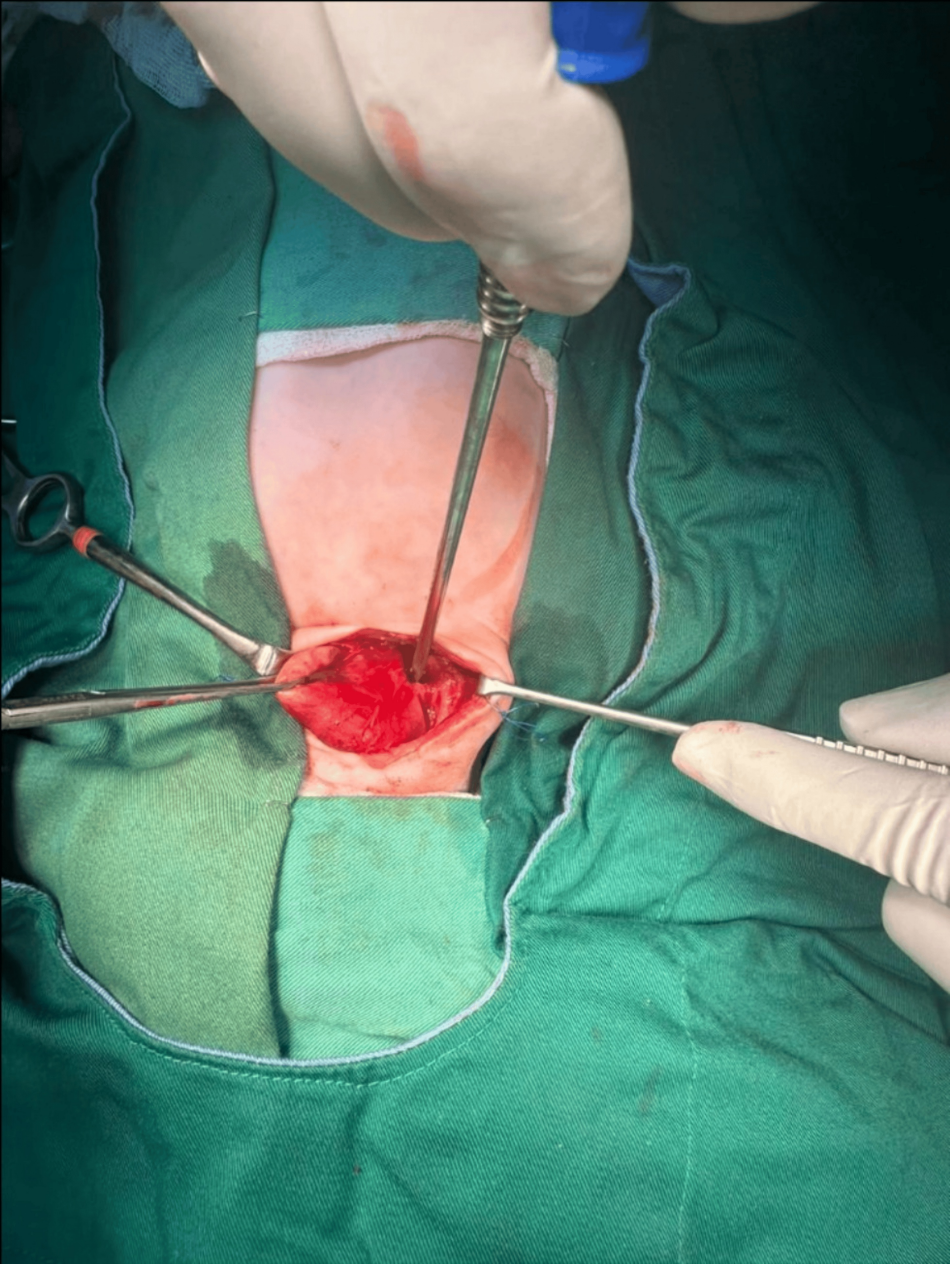
The image shows the intraoperative view of a neck surgery for the removal of a fourth branchial cleft cyst. Retractors are in place to expose the surgical field, while the surgeon uses fine instruments to carefully dissect around critical structures preserving nearby nerves and vessels.

**Figure 4:**
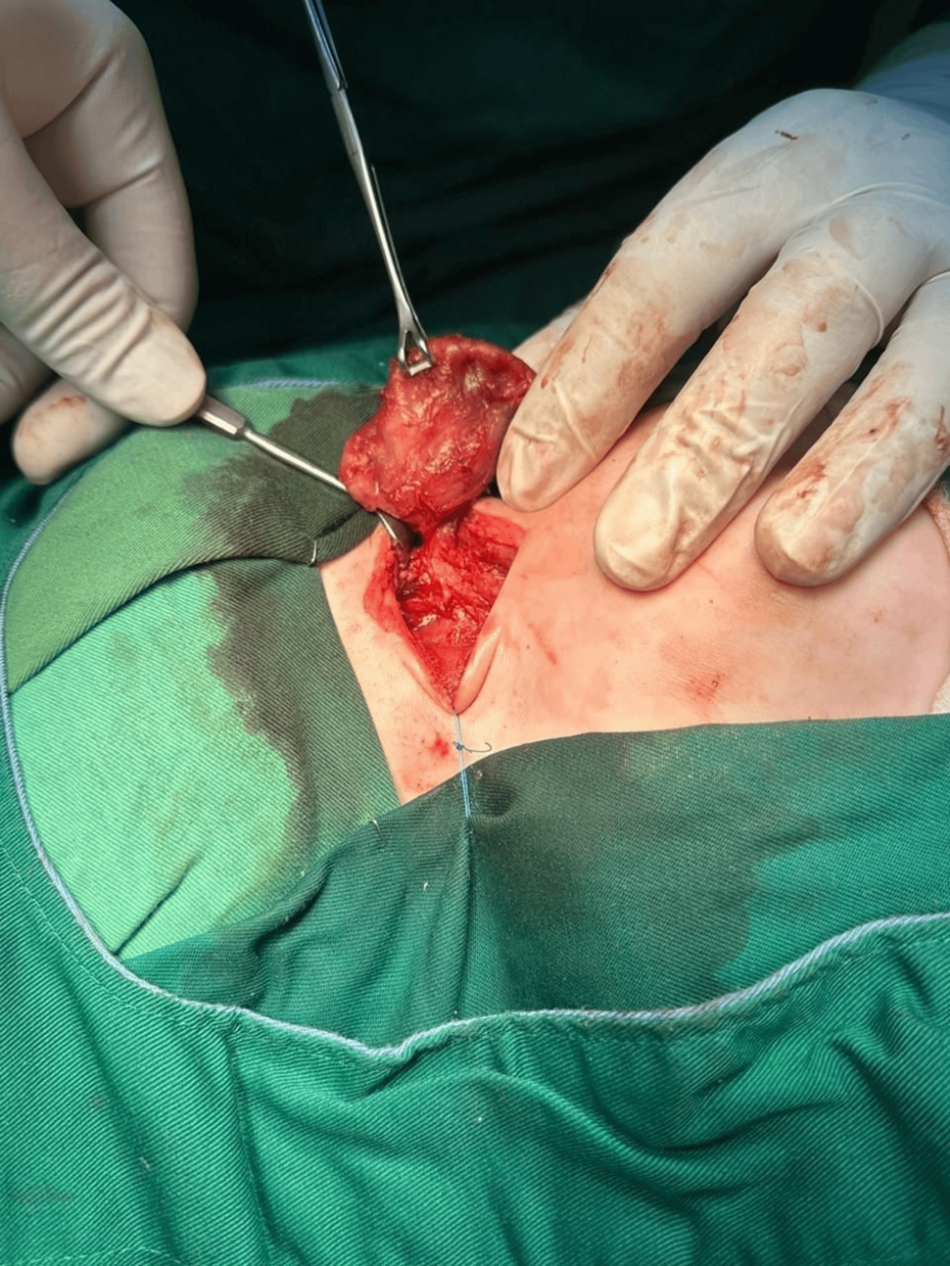
The image shows a close-up of a neck surgery where a mass is being carefully excised by the surgeon, using fine instruments to separate the tissue of interest.

After appropriate preparation and informed parental consent for the excision of cervical cyst with potential risks of bleeding, nerve injury, respiratory distress, anesthesia complication, and death, the procedure was performed under general anesthesia. The patient underwent surgical excision of the large cystic-solid mass located in the right carotid triangle, which appeared to originate from the pyriform sinus and extended into the upper mediastinum. This extension caused a shift of the mediastinal and cervical structures to the left and compressed both the esophagus and trachea (Figure 3 and Figure 4). While preserving all surrounding structures (recurrent laryngeal nerve, vagus nerve, carotid artery, esophagus, and trachea) and after opening the mass and draining the lymphatic fluid, the mass was completely excised followed by ligation of its root and securing hemostasis with no intraoperative complications. The specimen was sent for pathological examination.

The pathological results are detailed in Table 1.

**Table 1:** The pathology report.

Description	Comments
Gross description	The mass received in formalin, 6×3×3 cm cystic lesion. The cyst wall is 0.4-0.6 cm thick. It contains tan brown material.
Microscopic description	Representative sections are submitted. Sections show a fibrous cyst wall containing the thyroid tissue and an area of the parathyroid tissue. The cyst is lined partially by a stratified squamous epithelium; otherwise, the cyst lining is denuded/ulcerated with granulation tissue formation, presence of frequent macrophages, and occasional detached keratinizing squamous cells. The cyst wall additionally shows acute and chronic inflammation. There is no evidence of malignancy.
Diagnosis	Right lateral neck, excision: features consistent with fourth branchial cleft anomaly cyst. Negative for malignancy.

The patient experienced a full recovery after the procedure, with no complications observed during the postoperative period.

## Discussion

The branchial apparatus forms during the fourth to eighth weeks of gestation, consisting of five pairs of arches with cartilaginous centers (mesoderm), separated externally by four paired branchial clefts (ectoderm) and internally by four paired pharyngeal pouches (endoderm). This complex structure ultimately gives rise to the major anatomical features of the head and neck. Detailed information regarding the derivatives of the clefts, arches, and pouches, including the associated cranial nerves, is provided in Table 2 [11,3].

**Table 2:** Branchial arches' derivatives. CN: cranial nerve Reference: [3]

	Cleft	Arch	Pouch	Nerve
First branchial arch	External auditory canal	Mandible, incus, malleus, muscles of mastication, and CN V2 and V3	Eustachian tube, mastoid air cells	Maxillary nerve and mandibular branches of the trigeminal nerve (CN V2 and V3)
Second branchial arch	Cervical sinus	Stapes, the body of the hyoid, lesser horn of the hyoid, muscles of facial expression, and CN VII	Palatine tonsil	Facial nerve (CN VII)
Third branchial arch	Cervical sinus	Body of the hyoid, greater horn of the hyoid, superior constrictor muscles, internal carotid arteries	Thymus, piriform recess, inferior parathyroid glands	Glossopharyngeal nerve (CN IX)
Fourth branchial arch	Cervical sinus	Thyroid/cuneiform cartilages, CN X, aortic arch, right subclavian artery, various laryngeal muscles	Apex of the pyriform sinus, superior parathyroid glands	Vagus nerve (CN X), superior laryngeal nerve
Fifth and sixth branchial arches	None	Inferior pharyngeal constrictors, CN XI, various laryngeal muscles	Parafollicular C cells of the thyroid	Vagus nerve (CN X), recurrent laryngeal nerve

Branchial cleft anomalies such as cysts, sinus tracts, fistulas, and cartilaginous remnants can develop along the course of the first, second, third, or fourth branchial clefts due to improper closure during embryogenesis. These anomalies are typically associated with respiratory or squamous epithelium and can be inherited as autosomal dominant traits [11].

Fourth branchial cleft anomalies account for about 2% of all branchial cleft anomalies, making them extremely rare [3-6,10]. These anomalies are more commonly reported on the left side, with 93.6% occurring on the left, 6% on the right, and 0.5% bilaterally [8]. Approximately 9% of these anomalies are present during the neonatal period [11]. Cysts, which are remnants of sinuses without an external opening, typically manifest later in childhood compared to sinuses, fistulas, and cartilaginous remnants that develop during infancy [6]. The mean time from the onset of symptoms to diagnosis is reported to be five years [12].

In general, complete fistulas outnumber external sinuses, and both outnumber branchial cysts in childhood. Cysts predominate among adults. Fistulas and sinuses are usually identified by the discharge of fluids or purulent material from an aperture on the anterior border of the sternocleidomastoid [6]. Cysts are most commonly diagnosed as painless, compressible lateral neck lumps that may become uncomfortable and expand in size if infected.

Fourth BCCs in neonates can cause tracheal compression from fast expansion, resulting in dyspnea [6]. In infants and adults, the first clinical symptom of a fourth BCC is frequently an infectious mass. A recent assessment discovered that 42% of cases began as abscesses and 45% as thyroiditis.

Although fourth branchial arch cysts or sinuses are uncommon, it is essential to include them in the differential diagnosis of lateral neck masses. Other conditions to consider include carotid body tumors, paragangliomas, dermoid cysts, thyroglossal duct cysts, neurofibromas, hemangiomas, lipomas, teratomas, and lymph node metastases. Imaging techniques such as CT scans, ultrasounds (US), upper endoscopy, barium esophagography, and magnetic resonance imaging (MRIs) can help define the lesion and narrow the differential diagnosis. CT scans are most frequently used and can demonstrate fistulas in two-thirds of cases [6]. Upper endoscopy can help locate the pharyngeal opening, typically in the pyriform sinus or tonsillar fossae [10]. Barium esophagography has a 50-80% sensitivity for detecting third and fourth branchial fistulas. Fine-needle aspiration is necessary in adults to exclude metastatic carcinoma but is not required in children, and incisional biopsy should be avoided [6].

Congenital neck sinuses, cysts, cartilaginous remains, and fistulas should be treated with complete excision when there is no inflammation, as inflammation increases the risk of nerve injury and incomplete resection which should be avoided [6]. A case series of 18 BCCs indicated that surgical excision was effective, with no recurrences reported after 1-7 years [13]. Because of the rarity of these malformations, no standard surgical method has been documented; nonetheless, endoscopic management with diathermy of the fourth branchial cleft sinuses has become the standard of care due to its minimally invasive nature [14].

In this case, the neonate appeared with a solid cyst on the right side of her neck, which was classified as a fourth BCC, which is unusual given that neonates often have a fistula. Notably, her illness is not inherited. A CT scan revealed that the cyst had extended into the superior mediastinum, ruling out any possible diagnosis. The cyst's rapid development necessitated surgical surgery since it was compressing the patient's esophagus and trachea, causing eating and breathing issues, especially when she cried. The patient experienced a complete recovery following the procedure, with no complications during the postoperative period and no recurrence of the condition observed during follow-up.

## Conclusions

Fourth branchial cleft abnormalities are rare congenital conditions that are frequently misdiagnosed, particularly in newborns and toddlers, because of the rarity of the disorders, the low socioeconomic status of those affected, and the lack of proper diagnostic tools. This example illustrates the need to recognize, diagnose, and manage these rare defects to avoid problems. The total excision of the right BCC resulted in successful care, emphasizing the importance of early detection and action in comparable situations.
